# Supplementary material for: Comprehensive Approach to Genomic and Immune Profiling: Insights of a Real-World Experience in Gynecological Tumors
Source: Diagnostics (Basel). 2022 Aug 6;12(8):1903. doi: 10.3390/diagnostics12081903 (PMC9406465; doi:10.3390/diagnostics12081903)
Supplement: Supplementary file 1 [file diagnostics-12-01903-s001.zip › Table S2. List of antibodies.pdf]

Table S2. List of antibodies used to determine protein expression of gynecological biomarkers

Antibody name	Target Antigen	Vendor	Vendor identifier	Host organism	Clonality	Clone ID	Dilution	Assessment
MutL Protein Homolog 1 antibody	Human MLH1	Agilent	IR079	Mouse	Monoclonal	ES05	RTU	Absence/presence of tumor cell immunoreactivity
MutS Protein Homolog 2 antibody	Human MSH2	Agilent	IR085	Mouse	Monoclonal	FE11	RTU	Absence/presence of tumor cell immunoreactivity
MutS Protein Homolog 6 antibody	Human MSH6	Agilent	IR086	Rabbit	Monoclonal	EP49	RTU	Absence/presence of tumor cell immunoreactivity
Programmed death-ligand 1 antibody	Human PD-L1	Agilent	SK006	Mouse	Monoclonal	22C3	RTU	Combined positive score (CPS)
Postmeiotic Segregation Increased 2 antibody	Human PMS2	Agilent	IR087	Rabbit	Monoclonal	EP51	RTU	Absence/presence of tumor cell immunoreactivity
Anti-Human p53 Protein (Dako Omnis) antibody	Human p53	Agilent	GA616	Mouse	Monoclonal	DO-7	RTU	0-Normal expression, 1-overexpression, 2-cytoplasmatic expression, 3-complete absence
Anti-Human HER2 protein antibody	Human HER2	Agilent	SK00121-2	Rabbit	Monoclonal	Herceptest(R)	RTU	0-no staining, 1+incomplete membrane staining in <10% of tumor cells, 2+ strong complete membrane staining in ≤30% or incomplete in ≥10% and 3+ strong staining in >30% of tumor cells

CPS=Number of PD-L1 staining cells (tumor cells, lymphocytes, macrophages)/Total number of viable tumor cells x100
