# Supplementary material for: Comprehensive Approach to Genomic and Immune Profiling: Insights of a Real-World Experience in Gynecological Tumors
Source: Diagnostics (Basel). 2022 Aug 6;12(8):1903. doi: 10.3390/diagnostics12081903 (PMC9406465; doi:10.3390/diagnostics12081903)
Supplement: Supplementary file 1 [file diagnostics-12-01903-s001.zip › Table S5. Her2 by histological subtype_2.pdf]

Table S5. Alterations in ERBB2 by histological subtype

Patient	Tumor site	Histological subtype	ERBB2 NGS	ERBB2 IHC	ERBB2 FISH
11	Ovary	HGSC	WT	0+	-
14	Ovary	HGSC	CNV	3+	A
99	Ovary	HGSC	WT	2+	I
144	Ovary	HGSC	WT	1+	-
189	Ovary	HGSC	WT	1+	-
261	Ovary	HGSC	WT	0+	-
333	Ovary	HGSC	WT	0+	-
349	Ovary	HGSC	WT	0+	-
370	Ovary	HGSC	WT	0+	-
393	Ovary	HGSC	WT	2+	A
436	Ovary	HGSC	WT	0+	-
353	Ovary	CCC	WT	2+	NA
403	Ovary	CCC	SNV	1+	-
77	Ovary	Neuro	WT	0+	-
65	Uterus	Serous	CNV+SNV	2+	A
103	Uterus	Serous	WT	1+	-
163	Uterus	Serous	WT	1+	-
272	Uterus	Serous	WT	0+	-
375	Uterus	Serous	SNV	-	-
287	Uterus	CCC	WT	2+	A
176	Uterus	Endometrioid	WT	0+	-
201	Uterus	Endometrioid	SNV	2+	NA
245	Uterus	Endometrioid	WT	0+	-
325	Uterus	Endometrioid	WT	0+	-
437	Uterus	Endometrioid	WT	0+	-
237	Uterus	APA	WT	0+	-
388	Uterus	APA	WT	0+	-
36	Cervix	ADC	WT	0+	NA
121	Cervix	ADC	CNV	-	-
146	Cervix	Mucinous	WT	1+	-
444	Cervix	Mucinous	SNV	-	-
246	Cervix	SCC	WT	0+	NA

APA=atypical polypoid adenomyoma, HGSC=high-grade serous carcinoma, CCC=clear cell carcinoma, CNV=copy number variation, SCC=squamous cell carcinoma, SNV=single nucleotide variant, WT=wild-type, A=amplification, NA=non- amplification, I=indeterminate (non-evaluable due to specimen or assay failure)
