# Supplementary material for: Comprehensive Approach to Genomic and Immune Profiling: Insights of a Real-World Experience in Gynecological Tumors
Source: Diagnostics (Basel). 2022 Aug 6;12(8):1903. doi: 10.3390/diagnostics12081903 (PMC9406465; doi:10.3390/diagnostics12081903)
Supplement: Supplementary file 1 [file diagnostics-12-01903-s001.zip › Table S6. Costs testing strategy.pdf]

Table S6. Testing strategy, total cost and cost difference versus OCA NGS

	n=1	Ovarian HGSC patients, n=22	
Testing strategy	Testing cost	Total cost	Cost difference versus OCA NGS
TP53	150	3300	6512
BRCA NGS	404	8888	924
PALB2	150	3300	6512
RAD51	150	3300	6512
ERBB2	180	3960	5852
Total conventional testing	1034	22748	12936
OCA NGS	446	9812	-
PD-L1	161	3542	-
TILs	4	88	-
Total OCA NGS and immune testing	611	13442	3630

Costs are given in 2017 euros, HGSC=high-grade serous carcinoma, NGS=next-generation sequencing, OCA=oncomine comprehensive assay
