# Supplementary figures and images for: Comprehensive Approach to Genomic and Immune Profiling: Insights of a Real-World Experience in Gynecological Tumors
Source: Diagnostics (Basel). 2022 Aug 6;12(8):1903. doi: 10.3390/diagnostics12081903 (PMC9406465; doi:10.3390/diagnostics12081903)

(A)

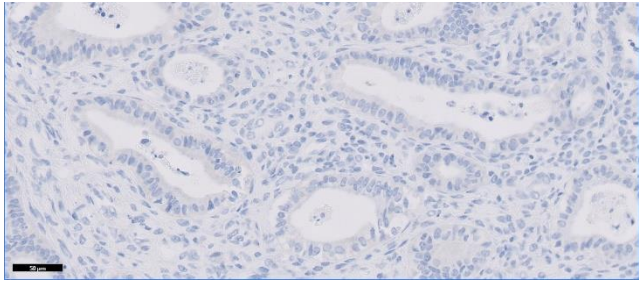

(B)

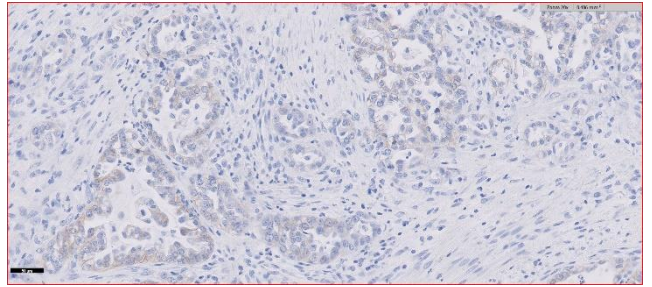

(C)

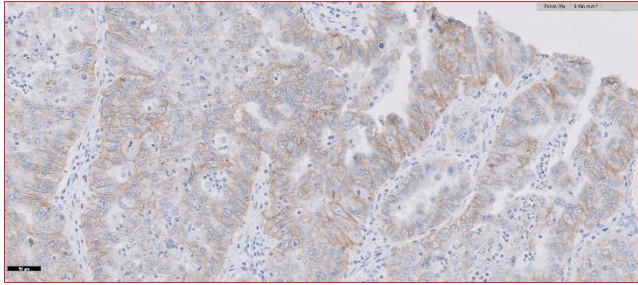

(D)

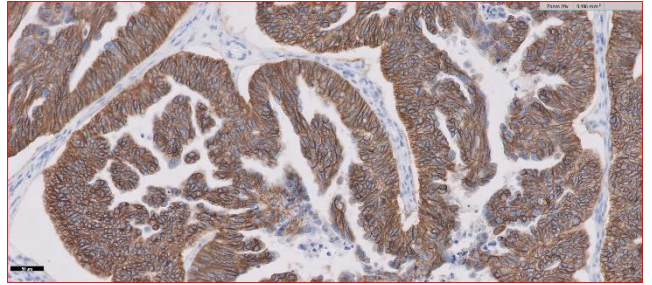

(E)

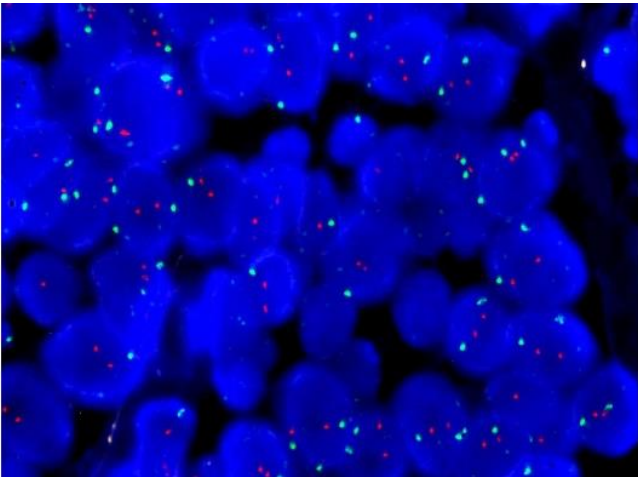

(F)

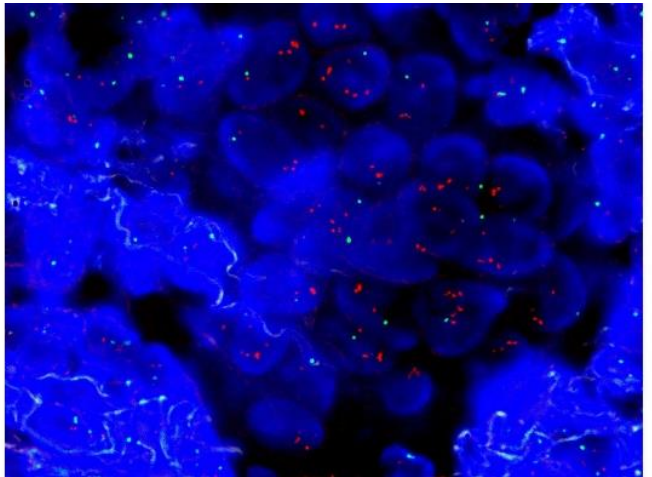

Supplement: Supplementary file 1 [file diagnostics-12-01903-s001.zip › Figure S1.pdf]

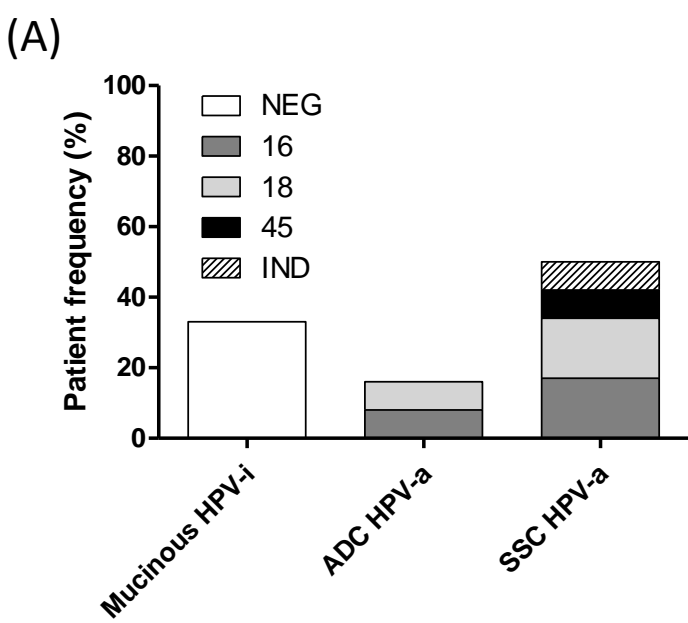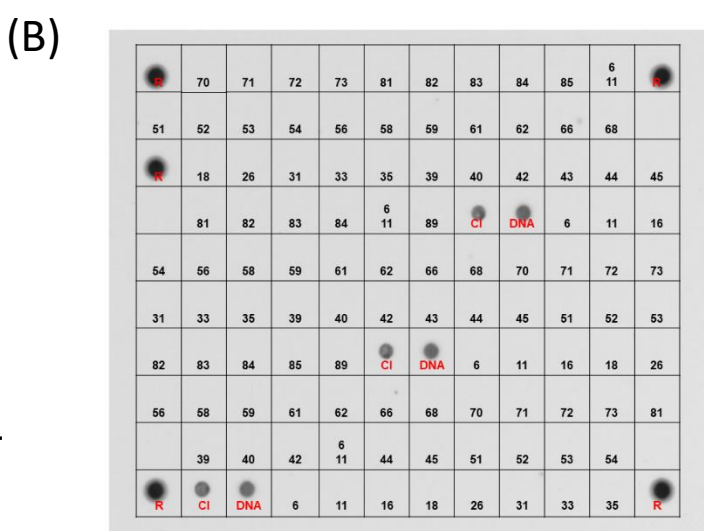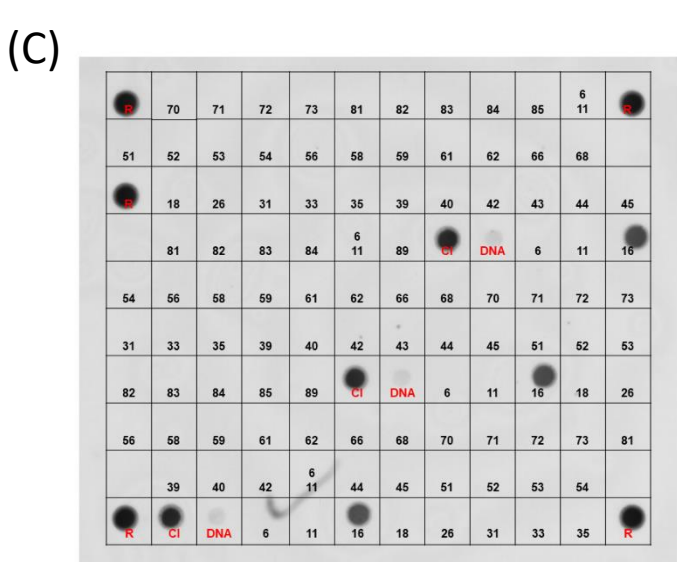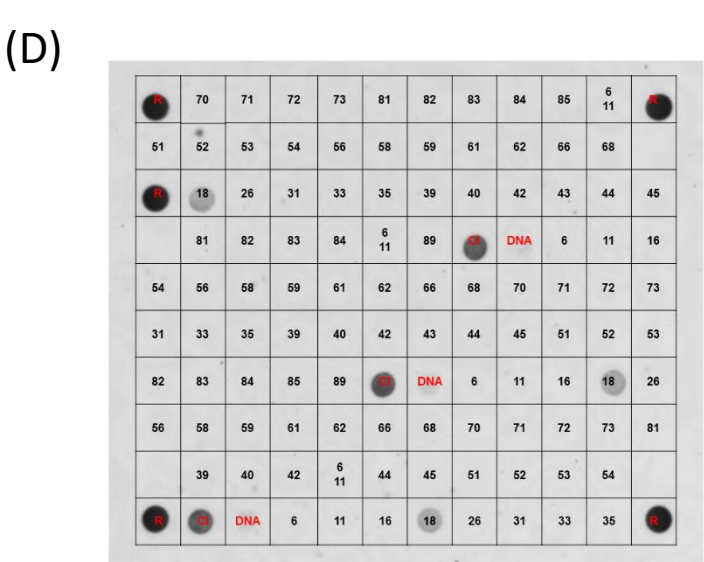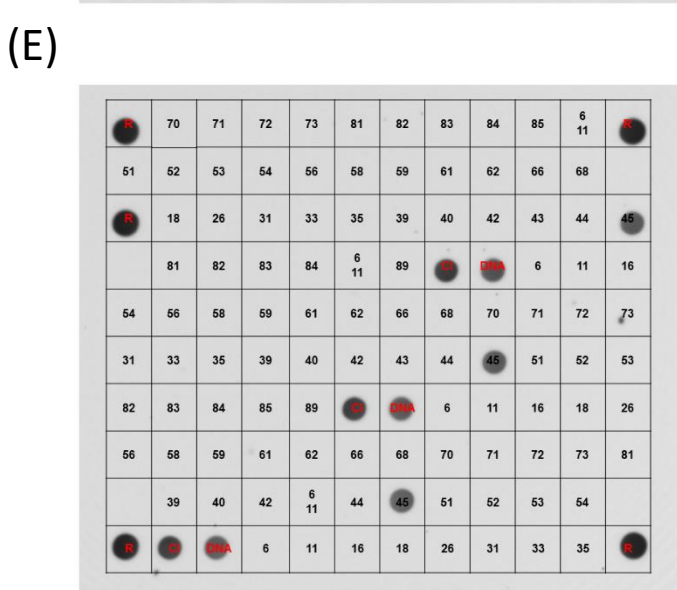

Supplement: Supplementary file 1 [file diagnostics-12-01903-s001.zip › Figure S2.pdf]
